# Binding Immunoglobulin Protein (BIP) Inhibits TNF‐α–Induced Osteoclast Differentiation and Systemic Bone Loss in an Erosive Arthritis Model

**DOI:** 10.1002/acr2.11060

**Published:** 2019-08-03

**Authors:** Mario M. Zaiss, Christopher Hall, Neil W. A. McGowan, Rebecca Babb, Vikesh Devlia, Sébastien Lucas, Sajeda Meghji, Brian Henderson, Aline Bozec, Georg Schett, Jean‐Pierre David, Gabriel S. Panayi, Agamemnon E. Grigoriadis, Valerie M. Corrigall

**Affiliations:** ^1^ Friedrich‐Alexander University Erlangen–Nürnberg (FAU) and Universitätsklinikum Erlangen Erlangen Germany; ^2^ King's College London London UK; ^3^ UCL‐Eastman Dental Institute University College London London UK; ^4^ Friedrich‐Alexander University Erlangen–Nürnberg (FAU) and Universitätsklinikum Erlangen, Erlangen, Germany, and Institute of Osteology and Biomechanics (IOBM) University Medical Center Hamburg‐Eppendorf Hamburg Germany

## Abstract

**Objective:**

The association between inflammation and dysregulated bone remodeling is apparent in rheumatoid arthritis and is recapitulated in the human tumor necrosis factor transgenic (*hTNF*tg) mouse model. We investigated whether extracellular binding immunoglobulin protein (BiP) would protect the *hTNF*tg mouse from both inflammatory arthritis as well as extensive systemic bone loss and whether BiP had direct antiosteoclast properties in vitro.

**Methods:**

*hTNF*tg mice received a single intraperitoneal administration of BiP at onset of arthritis. Clinical disease parameters were measured weekly. Bone analysis was performed by microcomputed tomography and histomorphometry. Mouse bone marrow macrophage and human peripheral blood monocyte precursors were used to study the direct effect of BiP on osteoclast differentiation and function in vitro. Monocyte and osteoclast signaling was analyzed by Western blotting, flow cytometry, and imaging flow cytometry.

**Results:**

BiP‐treated mice showed reduced inflammation and cartilage destruction, and histomorphometric analysis revealed a decrease in osteoclast number with protection from systemic bone loss. Abrogation of osteoclast function was also observed in an ex vivo murine calvarial model. BiP inhibited differentiation of osteoclast precursors and prevented bone resorption by mature osteoclasts in vitro. BiP also induced downregulation of CD115/c‐Fms and Receptor Activator of NF‐κB (RANK) messenger RNA and protein, causing reduced phosphorylation of the p38 mitogen–activated protein kinases, extracellular signal–regulated kinases 1/2 and p38, with suppression of essential osteoclast transcription factors, c‐Fos and NFATc1. BiP directly inhibited TNF‐α– or Receptor Activator of NF–κB Ligand (RANKL)–induced NF‐κB nuclear translocation in THP‐1 monocytic cells and preosteoclasts by the canonical and noncanonical pathways.

**Conclusion:**

BiP combines an anti‐inflammatory function with antiosteoclast activity, which establishes it as a potential novel therapeutic for inflammatory disorders associated with bone loss.

## Introduction

One of the many pathological facets of rheumatoid arthritis (RA) is the increased local and systemic loss of bone, leading to joint erosions and osteopenia, respectively. In RA, bone loss is exacerbated by the excessive production of inflammatory cytokines, especially tumor necrosis factor α (TNF‐α), in the local environment of the inflamed joint, which results in increased osteoclast differentiation and subchondral bone degradation as well as systemic bone loss 1, 2. The capacity for TNF‐α to drive osteoclastogenesis has been amply demonstrated in the human TNF‐α transgenic (*hTNF*tg) mouse model. These mice exhibit a rapidly developing, spontaneous, destructive arthritis with severe systemic osteopenia recapitulating the pathological features of RA 3. In our previous studies, we have demonstrated that binding immunoglobulin protein (BiP; or 78 kDa glucose‐regulated protein [GRP78]), a member of the heat shock protein 70 family, has prophylactic and therapeutic properties in the murine‐ collagen‐induced arthritis (CIA) model of RA when administered systemically as an extracellular protein 4, 5. Histological analysis of BiP‐treated arthritic mice showed complete preservation of the joint architecture with a lack of detectable damage to cartilage 4. However, whether BiP had any effects specifically on osteoclasts was not investigated. Because a major biomarker for extracellular BiP activity is the attenuation of TNF‐α activity 6, we investigated whether BiP had the potential to rescue the spontaneous arthritis in *hTNF*tg mice, with additional emphasis on the effects on osteoclasts both in vivo and in vitro.

Earlier human studies in vitro indicated that the primary effect of BiP was through monocytes, skewing their phenotype to that of a “deactivated monocyte,” inducing interleukin 10 (IL‐10) production and inhibiting TNF‐α 6. This is pertinent because peripheral blood (PB) monocytes represent a precursor population that differentiates into bone‐resorbing osteoclasts, dendritic cells (DCs), or tissue macrophages, depending on their niches, local cytokine environment, and the cell surface receptors expressed 7, 8. Indeed, we have reported that BiP treatment of PB monocyte–derived DCs skewed DC development toward indoleamine 2,3‐dioxygenase + (IDO^+^) anti‐inflammatory DCs and that direct cell contact between BiP‐treated IDO^+^ DC and autologous T cells, in the absence of further BiP, led to the induction of cytotoxic T‐lymphocyte–associated antigen 4 (CTLA‐4)^+^ regulatory T cells 9. In vivo, this may be part of the successful therapeutic action seen in the adoptive transfer studies in CIA, using mixed lymph node cells and splenocytes taken from BiP‐treated mice and injected into mice with ongoing CIA. Strikingly, these experiments confirmed that the therapeutic action of recombinant human BiP in patients with active RA was not reliant on the continued presence of the protein and suggested that one mechanism of action of BiP may be to induce differentiation of immunoregulatory T cells to correct the immunological imbalance induced by inflammation and, ultimately, to restore homeostasis 10, 11. This modulation of the immune cell subpopulations may account for the long‐lasting therapeutic effect following a single treatment. As BiP is detectable in human sera during both health and disease, it may assist in the restoration of homeostasis following an acute inflammatory incident. Indeed, in patients with RA, circulating levels of BiP are reduced compared with those of disease controls 11, which may explain, in part, why patients with RA have ongoing chronic inflammation.

The recognition of the importance of TNF‐α in the pathogenesis of RA has led to improved therapies 12. However, to reduce joint damage and disability, both inflammation and dysregulated bone metabolism need to be addressed. TNF inhibitors have a considerable therapeutic success in this respect 12. TNF‐α is a known activator of osteoclasts in inflammatory disease. However, although anti‐TNF biologics efficiently reduce inflammation, they are unable to inhibit systemic bone resorption. Thus, systemic bone loss is normally treated by additional antiresorptives, such as bisphosphonates. These offer long‐lasting protection from osteopenia but fail to control any concomitant inflammation and may be associated with serious adverse events 13. Therefore, the need remains for a novel treatment that delivers long‐term drug‐free relief from systemic bone loss, such as that found in RA.

In this study, a single intraperitoneal injection of BiP, administered at the onset of spontaneous arthritis in the *hTNF*tg mouse model, significantly alleviated disease by suppressing inflammation and protecting against bone loss. More importantly, BiP also inhibited osteoclast differentiation, which suggests that BiP has the unique potential to attenuate both the immunological parameters of inflammation as well as abnormal bone resorption. These functions suggest that BiP has considerable potential as a novel therapeutic in patients with active RA. Indeed, the recently completed Phase I/IIa clinical trial in RA patients confirmed that BiP was safe and showed some efficacy 10.

## Materials and Methods

### *hTNF*tg mice

*hTNF*tg mice (Tg197; genetic background C57BL/6) 14 develop a chronic inflammatory and destructive polyarthritis within 4 to 6 weeks after birth. A single intraperitoneal dose of BiP (10 μg/mouse prepared as described 4) or phosphate‐buffered saline (PBS) as vehicle control was injected at 5 weeks. Two independent experiments were performed each with four mice/group, and similar observations were obtained. All animal experiments were performed with the agreement of the ethical committee, and they followed local authorities at Erlangen University and the UK Home Office.

### Clinical assessment

Assessment of clinical parameters of arthritis (paw swelling, grip strength, cartilage degradation) was assessed semiquantitatively starting 1 week after initial treatment and subsequently at weekly intervals as described previously 15.

### Bone histomorphometry and histology

Histomorphometry was performed on sections of the paw or tibia 5 weeks after the beginning of the treatment for the *hTNF*tg mice. Histological analyses were performed as described previously 16. All quantifications were performed by digital image analysis (OsteoMetrics) using a microscope (Carl Zeiss) equipped with a digital camera.

### Microcomputed tomography

Microcomputed tomography (μCT) analysis was performed with a μCT40 scanner (Scanco) using the following parameters: voltage, 40 kV; X‐ray current, 250 μA; exposure time, 5000 ms/projection for 720 projections; matrix, 1024 × 1024; voxel size in reconstructed image, 9 μm. Images were analyzed with Scanco evaluation software for the following bone parameters at the metaphysis of the proximal tibia: ratio of bone volume to total volume, trabecular number, thickness, and separation.

### Cell culture

Murine bone marrow cells from 6‐ to 11‐week‐old CD1 mice or C57BL/6 mice were cultured overnight in Alpha MEM (MilliporeSigma) containing 10% batch‐tested fetal calf serum (FCS) and macrophage‐colony stimulating factor (M‐CSF) (50 ng/ml) (R&D Systems). After 24 hours, nonadherent cells were harvested and seeded either in culture dishes/plates or on dentine slices (3 × 10^4^ cells/6‐mm well), and cultured in the presence of M‐CSF (50 ng/ml) for 3 days followed by a further 3 to 4 days in 25 ng/ml M‐CSF and 5 ng/ml RANKL (R&D Systems), in the absence or presence of BiP (0.02‐20 μg/ml) as indicated. Tartrate‐resistant acid phosphatase (TRAP) staining for the evaluation of osteoclast differentiation was performed using a leukocyte acid phosphatase kit (MilliporeSigma). Osteoclasts were identified as TRAP‐positive cells with three or more nuclei. All experiments were repeated at least 6 times, and similar results were obtained in cells derived from both CD1 and C57BL/6 strains of mice.

For human studies, heparinized blood samples from healthy controls were collected after fully informed consent (Guy's Hospital Ethical Committee Ref: 01/05/01). PB monocytes were negatively selected from PB mononuclear cells (PBMCs) using immunomagnetic beads (Invitrogen). For differentiation studies, monocyte cultures were seeded into culture dishes or onto dentine discs (5 × 10^4^ cells/6‐mm well) and cultured in M‐CSF (50 ng/ml). After 4 days, cells were treated with M‐CSF (25 ng/ml) and RANKL (10 ng/ml) in the absence or presence of the indicated concentration of BiP (0.02‐20 μg/ml) for up to 10 days more. Osteoclasts were identified either by TRAP staining as detailed above, by tetramethylrhodamine staining, Phalloidin staining (MilliporeSigma) for F‐actin rings, or by immunolocalization for the vitronectin receptor (VnR) using the 23c6 antibody as described previously 17. Resorption pits were quantified following the removal of the cells and staining with Toluidine blue. In some experiments, BiP was added to mature osteoclasts at day 10 following the addition of RANKL. Resorption pits were quantified by image analysis using ImageJ. All experiments were repeated at least six times, and similar results were obtained.

THP‐1 cells (American Type Culture Collection [ATCC]) were grown in RPMI 1640 supplemented with heat‐inactivated 10% FCS and subcultured at 0.5 × 10^6^ cells/ml 24 hours before use. Cells were checked for their ability to bind BiP conjugated with fluorescein isothiocyanate and expression of RANK prior to exposure to BiP. Cells were pretreated for 1 hour with BiP and then stimulated with RANKL (50 ng/ml) (R&D Systems) or TNF‐α (10 ng/ml) (Peprotech) for 10 minutes.

### Quantitative reverse transcription polymerase chain reaction

Total RNA was isolated using TRIzol (Invitrogen). One microgram of total RNA was used for the first‐strand complementary DNA synthesis (Amersham Biosciences), which was then used for SYBR Green–based quantitative reverse transcription polymerase chain reaction (RT‐PCR) in triplicate according to the manufacturer's instructions. Gene expression was normalized to the housekeeping gene β‐actin. Specific primers: *cathepsin K*: Fwd 5’‐ATATGTGGGCCAGGATGAAAGTT‐3’; Rev 5’‐TCGTTCCCCACAGGAATCTCT‐3’, c‐*fms*: Fwd 5’‐ATGTCAAAGATCCGGCCCAC‐3’; Rev 5’‐GGTCAGTGATCAGACAGGGC‐3’, *RANK*: Fwd 5'TGGAACTCAGACTGCGAGTG‐3’; Rev 5’‐CCTTGTTGAGCTGCAAGGGA‐3’, β‐actin: Fwd 5‐TGTCCACCTTCCAGCAGATGT‐3’; Rev 5’‐AGCTCAGTAAC‐AGTCCGCCTAGA‐3’.

### Signaling studies and Western blotting

Purified human monocytes as above were cultured in the presence of 50 ng/ml M‐CSF followed by a further 48 hours in the absence or presence of 2 μg/ml BiP. Cells were then stimulated with 10 ng/ml RANKL for the times indicated, and whole‐cell lysates were prepared as previously described 18. Proteins were transferred to nitrocellulose and probed with the phospho‐specific extracellular signal–regulated kinases (ERKs) and p38 mitogen–activated protein kinases (MAPK) antibodies (Cell Signaling) or with antibodies for the transcription factors c‐Fos and NFATc1 (Santa Cruz Biotechnology). Equal protein loading was confirmed by blotting with antibodies against β‐actin or the appropriate total ERK or p38 as indicated. Rapidstep‐enhanced chemiluminescent reagent (Calbiochem) was used for visualization using a ChemiDoc XRS imager (Bio‐Rad).

### Flow cytometry and imaging flow cytometry

Osteoclast precursors were stained for cell surface expression of CD115/c‐Fms (BD Pharmingen) or RANK (Santa Cruz Biotechnology) as previously described 6 or for the intracellular presence of NF‐κB p65 after fixing in 4% paraformaldehyde in immunofluorescence (IF) buffer (0.5% bovine serum albumin, 0.01% sodium azide in PBS) for 5 minutes at room temperature and solubilizing the cells with 1% Triton X‐100 in IF buffer. Anti‐p65 (Cell Signaling Technology) was added for 45 minutes at 4°C, and phycoerythrin‐conjugated goat anti‐mouse IgG antibody (BD Pharmingen) was used as a secondary antibody. For NF‐κB nuclear translocation, M‐CSF–dependent osteoclast precursors or THP‐1 cells were cultured in the absence or presence of BiP (2 μg/ml), for NF‐κB p65 translocation, for 1 hour followed by 10 minutes of treatment with RANKL (10 ng/ml) or TNF‐α (10 ng/ml) or, for NF‐κB p52 translocation, for 4 hours with or without BiP (10 μg/ml) and/or RANKL (10 ng/ml). Nuclear translocation was visualized after staining with specific antibodies against p65 or p100/p52 (Cell Signaling Technology). Samples were analyzed by FACSCalibur (BD Biosciences) using Cellquest Pro software (BD Biosciences) or counterstained with 4′,6‐diamidino‐2‐phenylindole (DAPI) and analyzed by imaging flow cytometry (Imagestream X) using Ideas v.5 software. To visualize p52, nuclear translocation cells were viewed using an Eclipse Ni‐E inverted microscope (Nikon) at ×60 magnification and the A1 confocal imaging system (Nikon).

### Murine calvarial bone resorption assay

Bone resorption was assayed by measurement of calcium release from 5‐day‐old murine calvariae in culture 19, with five replicate cultures per treatment. After 24 hours, the medium was removed and replaced with medium containing RANKL (10 ng/ml) in the absence or presence of BiP as indicated. Replicate calvariae were cultured to determine the calcium release from unstimulated bone. After 48 hours, the media were removed and calcium content measured by automated colorimetric assay.

### Statistical analysis

All statistical analyses were performed with Student's *t* test (two groups) or with one‐way ANOVA followed by Tukey's test (more than two groups) and are represented as means ± SEM unless otherwise stated.

## Results

### BiP inhibited TNF‐α–induced inflammatory arthritis

**Figure 1:**
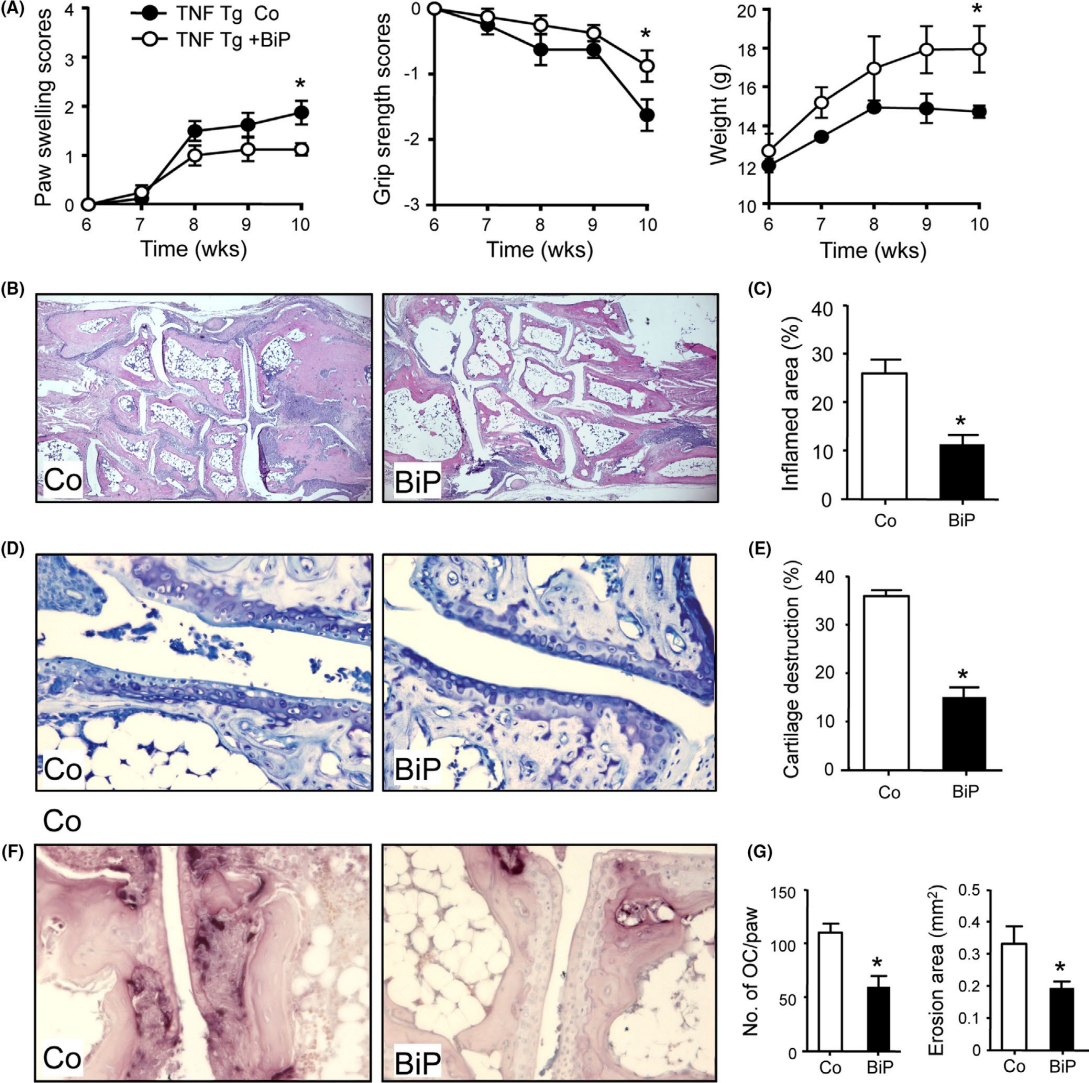
Binding immunoglobulin protein (BiP) ameliorates clinical and histological symptoms of arthritis in human tumor necrosis factor transgenic (*hTNF*tg) mice. (**A**) Clinical parameters, paw swelling, grip strength, and body weight measurements from BiP‐treated (+BiP) or phosphate‐buffered saline–treated (Co) *hTNF*tg mice (n = 4/group/experiment; data represent results of one out of two independent experiments). Histological sections of the tarsal joints of 10‐week‐old female *hTNF*tg mice and corresponding quantitative histomorphometric assessment of (**B**,** C**) synovial inflammation (haemotoxylin and eosin staining), (**D**,** E**) cartilage destruction (Toluidine blue staining), and (**F**,** G**) osteoclasts (tartrate‐resistant acid phosphatase [TRAP] staining), osteoclast numbers and bone erosions. *P < 0.05.

We first investigated the effects of extracellular recombinant BiP on *hTNF*tg mice that develop spontaneous joint inflammation and cartilage destruction typically between 4 and 6 weeks of age. A single intraperitoneal injection of BiP (10 μg) in 5‐week‐old *hTNF*tg mice ameliorated clinical parameters of disease, with significant decreases in progressive paw swelling and a concomitant rescue of grip strength and body weight by 10 weeks of age compared with the control (PBS) injected mice (Figure 1A). Comparative histological analysis by hematoxylin and eosin and Toluidine blue staining as well as quantification of the tarsal joints of BiP‐ or PBS‐treated *hTNF*tg mice revealed significantly decreased inflammation as well as decreased cartilage breakdown in the BiP‐treated mice (Figure 1B‐E). This bone‐protective effect was associated with fewer TRAP‐positive osteoclasts in the tarsal joints and an overall decrease in the bone erosion area (Figure 1F and G). These data indicate that BiP treatment attenuated the parameters of inflammatory arthritis, exerted an antiosteoclastogenic effect in the affected joints, and preserved joint architecture.

### BiP protected from TNF‐α–induced systemic bone loss

**Figure 2:**
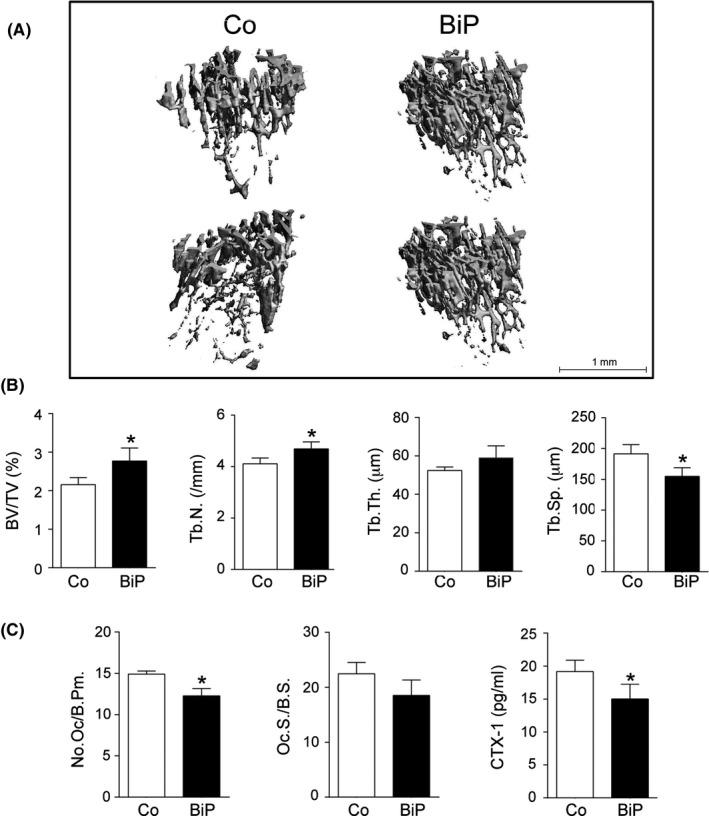
Binding immunoglobulin protein (BiP) protects from tumor necrosis factor (TNF)‐mediated systemic bone loss. Structural parameters of trabecular bone from the tibiae of 10‐week‐old BiP‐ or phosphate‐buffered saline–treated human TNF transgenic (*hTNF*tg) mice. (**A**) Representative micro‐computed tomography reconstruction of proximal tibiae from *hTNF*tg mice treated for 5 weeks in the absence or presence of 10 μg BiP showing increased trabecular bone mass in BiP‐treated animals. Trabecular bone parameters were quantified for (**B**) bone volume/total volume (BV/TV), trabecular number (Tb.N.), trabecular thickness (Tb.Th.) and trabecular separation (Tb.Sp.). (**C**) Quantitative histomorphometry of osteoclast numbers normalized to trabecular bone perimeter (No.Oc/B.Pm), osteoclast surface normalized to bone surface (Oc.S/B.S), and serum levels of C‐terminal telopeptide (CTX‐1). Data represent means ± SEM (n = 4 animals). *P < 0.05.

General osteopenia is a characteristic of human RA and is also observed in *hTNF*tg mice 3. TNF‐induced systemic bone loss was analyzed by assessing the microarchitecture of the proximal tibia, which is not directly exposed to the inflammatory environment of the joints. μCT analysis of proximal tibiae 5 weeks after a single BiP treatment revealed a marked preservation of bone mass (Figure 2A). Histomorphometric analysis of the proximal tibiae confirmed that a single injection of BiP significantly increased the trabecular bone volume (BV/TV) in *hTNF*tg mice, which was associated with an increased trabecular number (Tb.N.) and thickness (Tb.Th.) with a concomitant decrease in trabecular separation (Tb.Sp.) (Figure 2B). There were no significant changes in cortical bone parameters within the time frame of these experiments (data not shown).

The overall reduction in the number of osteoclasts observed by TRAP staining in the affected paws was also confirmed in the proximal tibiae by histomorphometry with a reduction in the number of osteoclasts per bone perimeter (N.Oc./B.Pm) and osteoclast surface per bone surface (Oc.S/B.S) in BiP‐treated *hTNF*tg mice compared with control mice (Figure 2C). This correlated well with a systemic reduction in serum type I collagen C‐terminal telopeptide, which is a marker of bone resorption (Figure 2C). Taken together, these data suggest that BiP has a therapeutic effect on both systemic and local bone destruction by inhibiting TNF‐induced joint inflammation as well as osteoclast differentiation and function.

### BiP directly suppressed murine and human osteoclast formation and function in vitro and ex vivo

**Figure 3:**
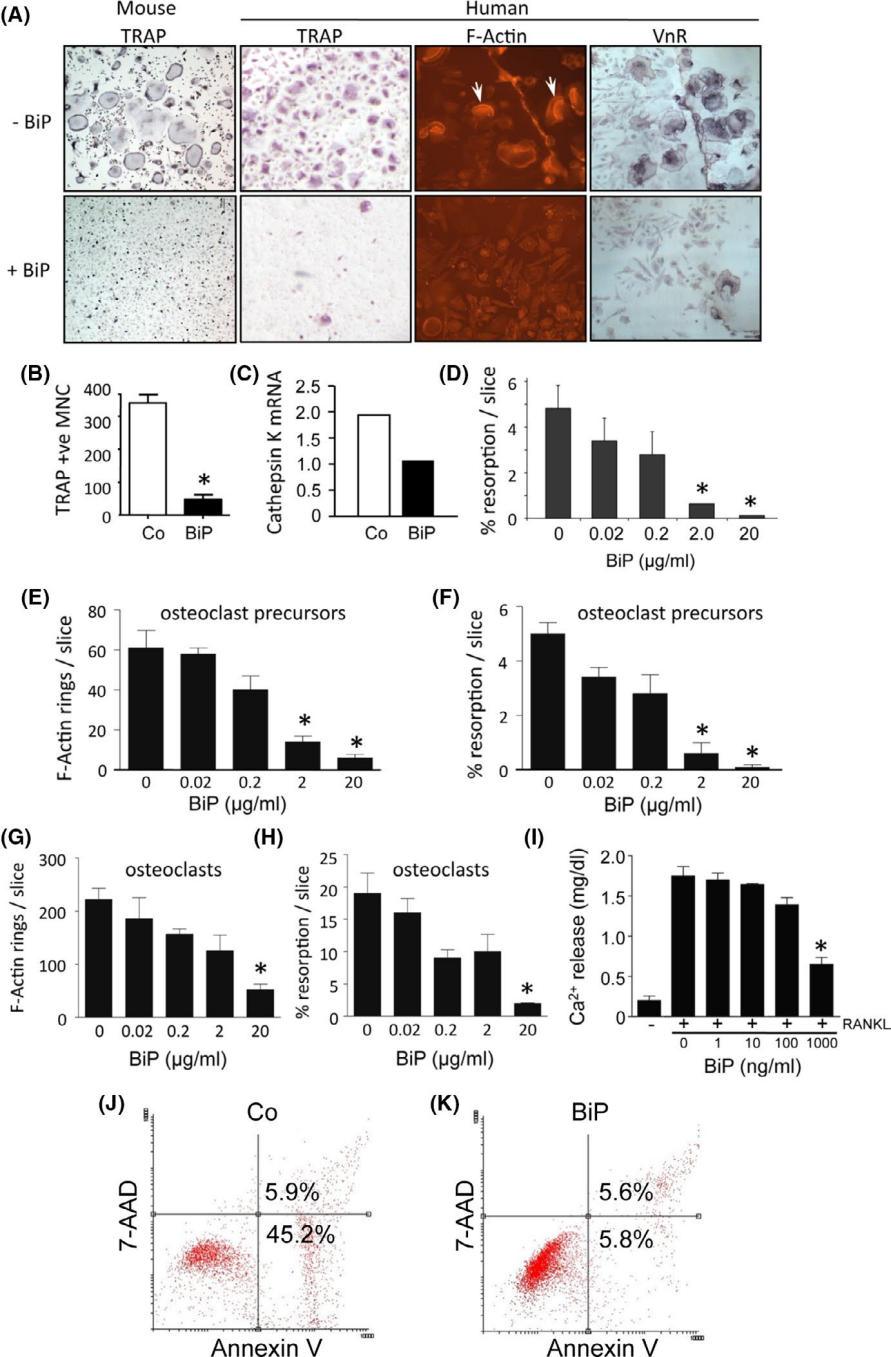
Binding immunoglobulin protein (BiP) specifically inhibits murine and human osteoclast formation and activity. (**A**) Representative images of the effects of continuous BiP (2 μg/ml) treatment on murine and human osteoclast differentiation as indicated. Osteoclasts are identified by staining for tartrate‐resistant acid phosphatase (TRAP) activity, F‐actin ring formation following phalloidin staining, and expression of vitronectin receptor (VnR; αvβ3 integrin) by immunocytochemistry using the specific 23C6 antibody. Phalloidin and VnR staining was performed on osteoclasts cultured on dentine slices. Quantification of the number of murine TRAP + ve cells (**B**) and quantitative polymerase chain reaction analysis of cathepsin K expression (**C**) following BiP (2 μg/ml) treatment. (**D**) Dose‐response analysis of murine osteoclast resorption following BiP treatment. (**E**) Dose‐dependent effects of BiP on the number of F‐actin ring‐positive human osteoclasts cultured on dentine; *P < 0.007, and corresponding resorptive activity (**F**) *P < 0.004, when BiP was added continuously from the start of culture (osteoclast precursors). (**G**) Dose effects of BiP on the number of F‐actin ring‐positive human osteoclasts; *P < 0.02, and (**H**) corresponding resorptive activity; *P < 0.04, when BiP was added to late‐stage cultures enriched with mature osteoclasts. (**I**) Ca^2+^ release representing resorption from intact murine calvarial bones cultured in the presence of receptor activator of NF‐κb ligand (RANKL) (10 ng/ml) and the indicated concentrations of BiP (1‐1000 ng/ml). *P < 0.05. Apoptosis was assessed in control (**J**) or BiP‐treated (**K**) preosteoclasts by flow cytometry using 7‐aminoactinomycin D and Annexin V staining, showing a reduction in the % early apoptotic cells (45.2% vs 5.8%). No differences in any in vitro parameters were observed between male and female donors/mice.

The previous data showed that BiP treatment decreased osteoclast numbers and resorptive activity in both the paw and tibia. This suggested a potential direct effect on osteoclasts, although it was difficult to assess from the in vivo studies whether this was secondary to the established anti‐inflammatory properties of BiP. To address this, we investigated whether BiP acts directly on osteoclast precursors or on mature osteoclasts in vitro using murine bone marrow and human PBMC‐derived osteoclasts. Continuous exposure of murine M‐CSF–dependent bone marrow–derived osteoclasts to BiP caused an inhibition in the number of TRAP‐positive osteoclasts and expression of the osteoclast marker cathepsin K (Figure 3A‐C). Analysis of the osteoclast resorptive function showed that BiP treatment caused a dose‐dependent inhibition in the percentage of resorbed area on a mineralized dentine substrate (Figure 3D). Exposure of human M‐CSF–dependent PBMCs to BiP similarly caused a dose‐dependent inhibition in the formation of osteoclasts as demonstrated by a decrease in the number of cells showing the characteristic F‐actin ring structure of TRAP‐positive multinucleated cells as well as a decrease in VnR (αvβ3 integrin)‐positive cells (Figure 3A and E). The reduction in human osteoclast numbers resulted in a concomitant dose‐dependent decrease in bone resorption as measured by lacunar formation on dentine slices (Figure 3F). To investigate whether BiP also acted on cultures enriched in mature osteoclasts, pulse experiments were performed in which BiP was added in late‐stage cultures after osteoclast differentiation had occurred on dentine slices. BiP caused a dose‐dependent decrease in the number of active F‐actin ring‐positive osteoclasts and inhibited the resorptive ability of mature osteoclasts (Figure 3G and H). For independent confirmation of the putative direct antiresorptive effects of BiP that are independent of inflammation, we used an ex vivo murine calvarial resorption model. Addition of BiP to intact calvariae activated with RANKL significantly reduced resorption as measured by the release of calcium ions into the culture supernatant (Figure 3I). Finally, the decrease in osteoclast numbers was not due to cell death or apoptosis. Annexin V staining showed that BiP treatment did not reduce cell viability, and indeed, cells cultured with BiP were less prone to apoptosis than control cells (Figure 3J and K). Taken together, these results suggest that BiP suppresses the differentiation of osteoclast precursors as well as the function of mature osteoclasts both in vitro as well as ex vivo in a manner that is independent of its anti‐inflammatory properties.

### BiP suppressed osteoclast differentiation signaling pathways

**Figure 4:**
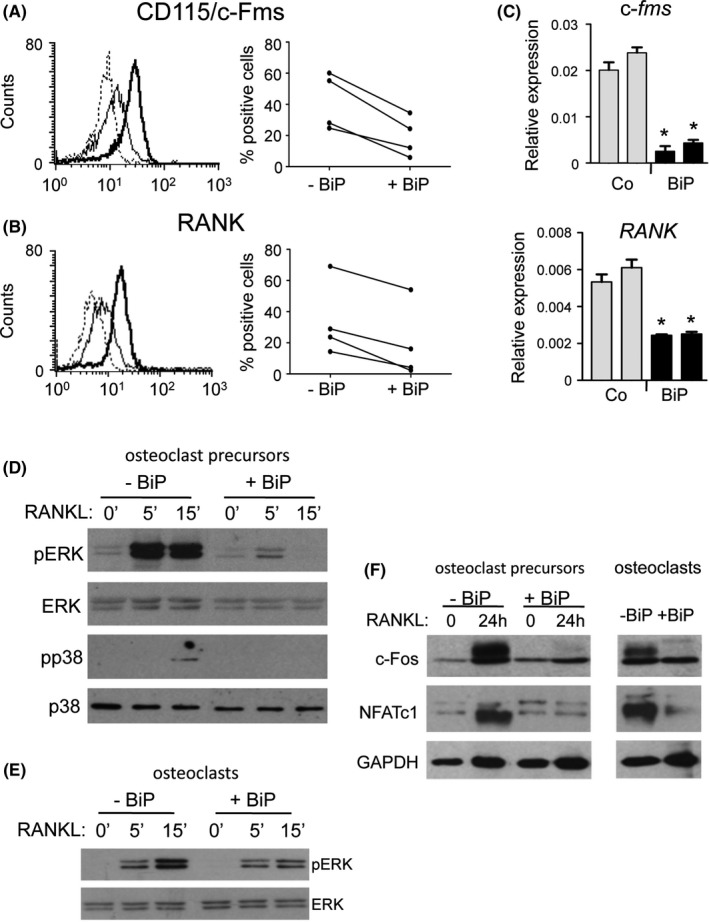
Binding immunoglobulin protein (BiP) downregulates CD115 and receptor activator of NF‐κb (RANK) cell surface expression and downstream signaling in human osteoclast precursors. Flow cytometry analysis of expression of CD115/c‐FMS (**A**) and RANK (**B**) protein levels by macrophage‐colony stimulating factor (M‐CSF)‐dependent human osteoclast precursors cultured in the absence or presence of BiP (2 μg/ml) for 48 hours. Representative samples showing mean fluorescence intensity: dotted line‐negative isotype control; heavy solid line; control‐BiP treatment; light solid line: +BiP (n = 4). (**C**) Quantitative polymerase chain reaction analysis of c‐*fms* and RANK expression following 48‐hour treatment of murine M‐CSF–dependent osteoclast precursors with BiP (2 μg/ml). Data show the means ± SEM of duplicate experiments using specific primers and normalized to β‐actin. *P < 0.05. (**D**) Western blot analysis showing expression of pERK and pp38 in human osteoclast precursors in response to RANK ligand (RANKL) (10 ng/ml) for the indicated times in cells cultured in the absence or presence of BiP (2 μg/ml, 48h). (**E**) Expression of RANKL‐induced pERK in cultures containing mature human osteoclasts cultured in the absence or presence of BiP (2 μg/ml, 48 hours). (F) Western blot analysis showing expression of c‐Fos and NFATc1 in response to RANKL (10 ng/ml) in osteoclast precursors and mature osteoclasts treated in the absence or presence of BiP (2 μg/ml). Total ERK and p38 proteins, and glyceraldehyde 3‐phosphate dehydrogenase (GAPDH) were used as loading controls as indicated. *P < 0.01.

To investigate the mechanisms underlying the inhibition of osteoclastogenesis by BiP, we analyzed specific cytokine signaling and downstream signaling pathways known to be essential for osteoclast differentiation. Flow cytometric analysis of CD115/c‐Fms and RANK, the receptors for M‐CSF and RANKL, respectively, in human PB–derived M‐CSF–dependent osteoclast precursors revealed that 48‐hour treatment with BiP downregulated the expression of CD115 by 63% ± 16% (range of inhibition: 43% to 79%) (Figure 4A). Similarly, 48‐hour treatment with BiP inhibited RANK protein expression by 51% ± 29% (range of inhibition: 22% to 90%) (Figure 4B). The inhibition of c‐Fms and RANK expression was also observed at the messenger RNA level, as quantitative PCR analysis showed significant reductions in c‐*fms* and *RANK* RNA (Figure 4C). To address whether the decrease in receptor expression resulted in reduced responsiveness to osteoclastogenic cytokines, we analyzed the effect of BiP treatment on RANKL‐dependent MAPK signaling. Pretreatment of human PBMC–derived osteoclast precursors for 48 hours with BiP markedly suppressed RANKL‐induced ERK and p38 phosphorylation compared with untreated cells (Figure 4D). Further examination of RANKL responsiveness in late‐stage cultures similarly revealed that RANKL‐induced pERK levels were also attenuated in cultures enriched in mature osteoclasts following BiP treatment (Figure 4E).

### BiP suppressed osteoclast transcription factors

We next investigated the effect of BiP on the expression of the transcription factors c‐Fos and NFATc1, which are essential for osteoclast differentiation and lie downstream of RANK and TNF‐α signaling in osteoclast precursors and monocytes. Preincubation of human osteoclast precursors with BiP for 48 hours greatly reduced the activation of the c‐Fos protein following RANKL treatment (Figure 4F). RANKL stimulation of NFATc1, a c‐Fos target gene 20, was similarly blocked in osteoclast precursors pretreated with BiP when compared with control cell lysates (Figure 4F). Mature osteoclast cultures treated with BiP also showed a marked decrease in the endogenous expression of both c‐Fos and NFATc1 transcription factors (Figure 4F).

**Figure 5:**
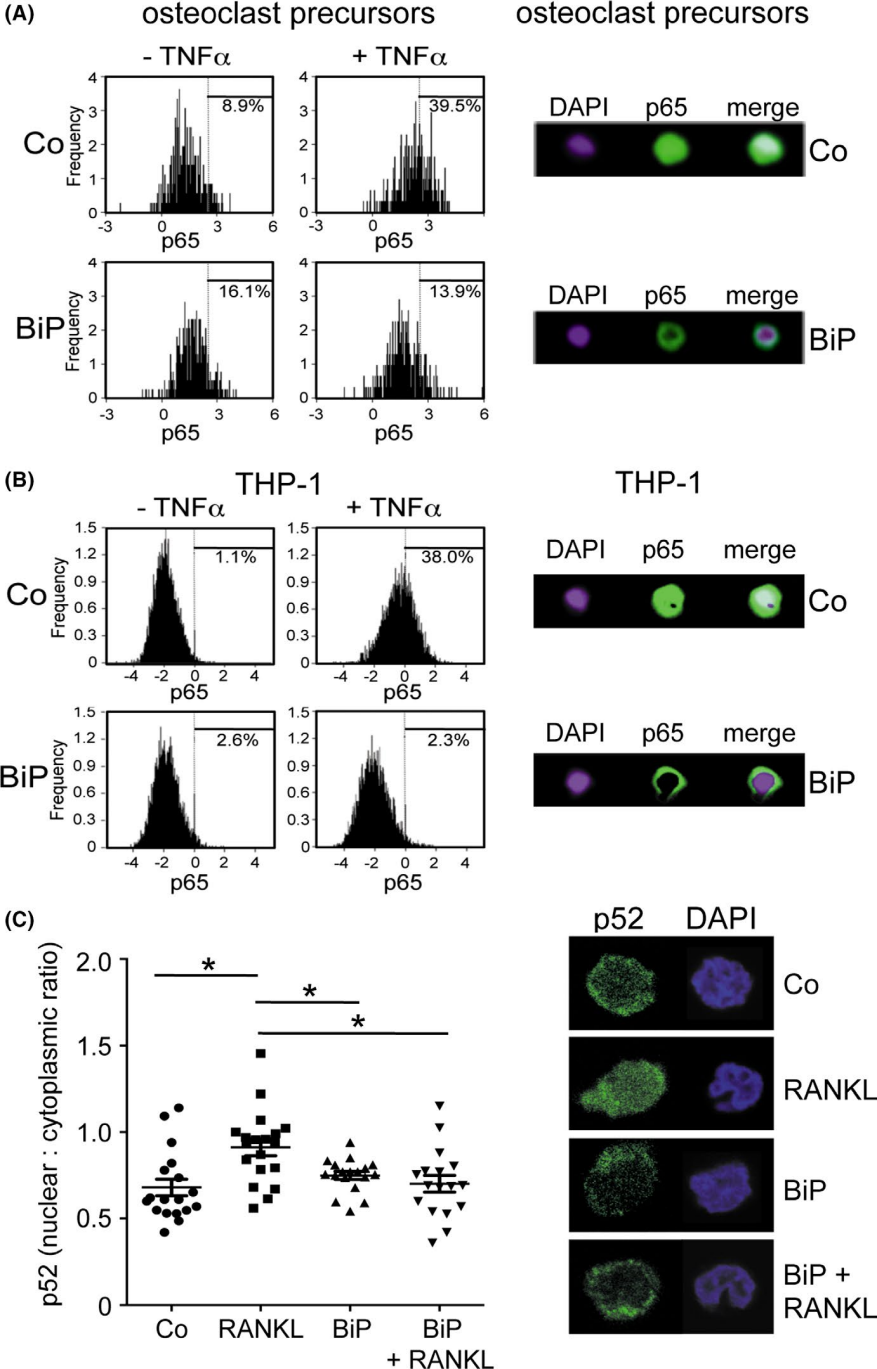
Binding immunoglobulin protein (BiP) inhibits nuclear translocation of NF‐κB p65 and p52 in osteoclast precursors and THP‐1 monocytes following TNF‐α and RANKL stimulation. M‐CSF–dependent (**A**) human osteoclast precursors or (**B**) THP‐1 cells were pre‐treated for 1 hour in the absence (Co) or presence of BiP (10 μg/ml) (**A**,** B**) and then stimulated with TNF‐α (10 ng/ml) for 10 minutes. (**C**) Preosteoclast were cultured in the presence or absence of BiP (10 μg/ml) with or without RANKL (50 ng/ml) for 4 hours. Cells were fixed and processed for flow cytometry, imaging flow cytometry, or confocal microscopy as described in the Methods section following staining for NF‐κB p65 (**A**,** B**) or p52 (**C**) with DAPI counterstain. Panels on the right of each figure section show confocal images of nuclear translocation of p65 and p52 in a representative single cell, showing the absence of nuclear translocation in BiP‐treated cells. *P < 0.01, n = 3.

Whether BiP could similarly inhibit TNF‐α–induced NF‐κB signaling, which is also essential for osteoclastogenesis, was investigated next in human monocyte–derived osteoclast precursors as well as in the monocyte cell line THP‐1, which shows TNF responsiveness. Imaging flow cytometry demonstrated that 1 hour of BiP treatment inhibited the TNF‐α–induced nuclear translocation of p65 NF‐κB in both osteoclast precursors and THP‐1 cells (Figure 5A and B). Similar results were obtained in response to RANKL stimulation (data not shown). Because RANKL also stimulates cells via the noncanonical NF‐κB pathway, we investigated the nuclear translocation of p52 NF‐κB following RANKL stimulation. Confocal microscopy and image analyses showed that although RANKL stimulated efficient nuclear translocation of p52 in untreated cells, this was inhibited by BiP pretreatment (Figure 5C). These results suggest that BiP blocks both canonical as well as noncanonical NF‐κB signaling in monocytes and osteoclast precursors following TNF‐α and RANKL treatment.

Taken together, these data demonstrate that treatment of monocytes and osteoclast precursors with BiP reduces M‐CSF– and RANKL‐induced signal transduction and activation of the essential osteoclastogenic transcription factors NF‐κB, c‐Fos, and NFATc1, thereby providing insights into the mechanisms whereby BiP could inhibit osteoclast differentiation and resorptive activity in vivo.

## Discussion

In this report, we have shown that BiP treatment of *hTNF*tg mice had extended therapeutic efficacy with protection not only from inflammatory arthritis but also bone loss, both systemic and local, following a single administration at the onset of disease.

TNF‐α is acknowledged as a major driver of disease pathology in RA and therefore the ability of anti‐TNF agents to protect from bone loss has been keenly investigated. It is well‐established that the immune response has a regulatory role in bone remodeling and that uncontrolled chronic inflammation is a critical part of disease‐induced bone loss 1, 21. In addition to RANKL, cytokines (TNF‐α, IL‐6, IL‐4, IL‐10, and TGF‐β) and immune cells (CD4^+^CD25^hi^ and CTLA‐4^+^ regulatory T cells) are complicit in the positive and negative regulation of osteoclastogenesis and may act independently of RANK/RANKL 22, 23, 24. It is clear that although targeting such immune and inflammatory pathways, especially TNF signaling, is currently used in the clinic, the reversal of systemic bone loss and structural changes in RA by anti‐TNF agents alone does not appear to prevent osteopenic disorders independently of inflammation. As a result, current combination therapies may incorporate drugs that specifically target osteoclast activity, such bisphosphonates and anti‐RANKL (denosumab) 25, 26, 27, 28, 29. This highlights one important difference between existing therapeutics and the mode of action of the immune modulator, BiP, which fulfils a dual role by targeting both the inflammatory component of RA as well as the systemic bone loss by acting additionally on the osteoclast lineage, thus accentuating the novelty of BiP as a potential therapeutic.

In vitro studies revealed that BiP can directly inhibit osteoclast differentiation. Addition of BiP to preosteoclast cultures downregulated two critical cell surface receptors, CD115/c‐FMS and RANK, although the mechanisms of this inhibition are not yet clear. CD115 has already been investigated as a potential target for therapy in RA through the use of specific blocking anti‐CD115 monoclonal antibody 30, 31. M‐CSF/CD115 ligation has been shown to upregulate RANK; therefore, the downregulation of RANK observed in BiP‐treated cultures could be mediated in part by decreased M‐CSF signaling 32. This was confirmed in BiP‐treated osteoclast precursor cultures that showed strongly suppressed RANKL‐induced phosphorylation of ERK and p38 MAPKs, which would normally be rapidly phosphorylated following c‐FMS and RANK receptor activation 33. How BiP interferes with downstream RANK activation is not yet known, but is likely to involve the TRAF family adaptor proteins that are essential for mediating RANK‐mediated signaling in osteoclasts 34.

The inflammatory cytokine TNF‐α induces NF‐κB activation and thereby augments the production of other proinflammatory cytokines, which also act through NF‐κB. Both the canonical and noncanonical pathways lead to NF‐κB translocation and have a degree of redundancy, counterregulation, and ligand selectivity. Both TNF‐α and RANKL can act via the classical canonical pathway, allowing nuclear translocation of NF‐κB p65. Additionally, RANKL can act via the noncanonical route, which is driven by ubiquitination and processing of the cytoplasmic inhibitor NF‐κB p100 to form NF‐κB p52 34. Strikingly, we observed that BiP inhibited nuclear translocation of NF‐κB by both these pathways in response to both TNF‐α and RANKL. Together with the already known anti‐inflammatory properties of extracellular BiP, this may explain the long‐lasting effects of BiP on TNF‐α–driven pathology. The inhibition of the essential osteoclast transcription factors c‐Fos and NFATc1 also confirm that the inhibitory effects of BiP are manifested across the entire signal transduction cascade that drives osteoclastogenesis. The direct osteoclast inhibitory functions of BiP in vitro were manifest at two levels, inhibition of osteoclast precursors as well as mature osteoclasts characterized by inhibition of F‐actin rings and/or expression of the osteoclast markers TRAP, VnR, and cathepsin K.

Taken together, these results predict that BiP will have therapeutic value in human localized and systemic osteolytic disease because of its dual action on mature osteoclast function and differentiation in addition to its anti‐inflammatory properties. Whether BiP can inhibit osteoclasts independently of its anti‐inflammatory effects is an interesting issue that is yet to be resolved as it may be context dependent. One possible candidate for mediating an indirect effect of BiP on osteoclasts in vivo might be IL‐10, which is an established osteoclast inhibitory cytokine and which we have shown to be stimulated by BiP in PBMCs in vitro 6. However, our data show unequivocally that BiP has direct effects on osteoclast precursors and mature osteoclasts in vitro and also inhibits osteoclastic resorption in ex vivo calvarial organ culture, which is a noninflammatory model. Therefore, it is tempting to speculate that BiP might indeed have direct effects on osteoclasts that are separate from its anti‐inflammatory effects; however, we have not yet demonstrated direct effects of BiP on osteoclasts in our study as the *hTNF*tg in vivo model is not able to separate the two processes. Indeed, it remains of prime importance to investigate whether BiP has independent effects on the osteoclast lineage in vivo in view of the current notion that there are molecular differences between subsets of osteoclasts associated with either normal bone resorption/turnover or those associated with different pathologies, such as RA or even cancer‐induced bone loss 35, 36, 37, 38. Nevertheless, as regards RA and the *hTNF*tg model in our study, whether BiP is acting directly or indirectly on osteoclasts does not diminish its potential as a novel therapeutic.

How BiP affects its target cell populations remains to be determined as a putative receptor for BiP has not yet been identified. Interestingly, as BiP belongs to the heat shock protein 70 (HSP70) family and shows a 60% to 70% homology with this molecule, it does not bind any of the multiple receptors identified for HSP70 11. This adds weight to the fact that although HSP70 extracellular activity tends to be proinflammatory, BiP has profound anti‐inflammatory effects.

As a stress protein, endogenous intracellular BiP is upregulated during cell stress and associated with antiapoptotic and cell‐protective functions 39. However, these properties of the intracellular protein are strikingly different from those of cell‐free BiP. Thus, BiP has been defined as a moonlighting protein, with protein in the intracellular or extracellular compartments having distinct and disparate functions 40. This was evident in our previous studies, which indicated there was no advantage to delivering exogenous BiP at the focus of inflammation, where BiP is already overexpressed 41. Rather, a systemic delivery that allowed readjustment of the immune system with upregulation of anti‐inflammatory cytokines 6 and induction and expansion of regulatory T cells and tolerogenic DCs 9, 11 is preferred. These changes underlie the ability of BiP to modulate the immune response, thus providing potentially well‐tolerated and long‐term therapy 11, 42, 43. In contrast, manipulation of the *grp78* gene revealed that inflammation increased with greater endogenous BiP production, possibly caused by the antiapoptotic function of intracellular BiP 44. Similarly, in a murine model of osteoporosis, Hino et al showed that inducing endogenous BiP protected against declining bone production, which they attributed to effects on osteoblasts; however, they did not explore the effect on osteoclast differentiation or function 45. In contrast, our studies show that extracellular BiP clearly provides significant anti‐inflammatory and antiresorptive effects even under the unnatural conditions of excessive TNF‐α production, such as in the *hTNF*tg mouse.

RA patients have reduced levels of circulating BiP 11, which may explain, in part, why patients with RA have ongoing chronic inflammation. Using the *hTNF*tg spontaneous model of arthritis in vivo, combined with in vitro effects on osteoclasts, our data clearly demonstrate that BiP has additive, likely synergistic, anti‐inflammatory, and antiosteoclast activity. In the recent Phase I/IIA clinical trial in RA, BiP was shown to be safe and well tolerated, and biomarker analysis showed considerable anti‐inflammatory activity with clinical benefit 10. Whether this extends to protection from bone loss in RA will be examined in the Phase IIB clinical trial.

## Author Contributions

Drs. Zaiss, Bozec, Schett, David, Panayi, Grigoriadis, and Corrigall prepared and revised the manuscript and provided the final approval. Drs. Grigoriadis and Corrigall are the final guarantors.

### Study design and conception

Zaiss, Bozec, Schett, David, Panayi, Grigoriadis, Corrigall.

### Acquisition of data

Zaiss, Hall, McGowan, Babb, Devlia, Lucas, Meghji, Henderson, Bozec, David, Grigoriadis, Corrigall.

### Analysis and interpretation of data

Zaiss, Hall, Bozec, Schett, David, Panayi, Grigoriadis, Corrigall.
